# Hepatic Colic

**Published:** 1891-04-25

**Authors:** 


### THE PRACTITIONER'S MIRROR AND RETROSPECT.

[*The Editor will be glad to receive offers of co-operation and contributions from members of the profession. All letters should be addressed to* THE EDITOR, THE LODGE, PORCHESTER SQUARE, LONDON, W.]

### MEDICINE.

### HEPATIC COLIC.

The treatment of hepatic colic is discussed by Professor Germaine Sée in a recent number of *La Médicine Moderne.* As he remarks, the chief indication is, of course, to promote the expulsion of the offending gall-stone, and to attain this end Professor Sée advocates the employment of drugs which act by increasing the secretion of bile. The very intense pain, however, generally calls for immediate relief, and for this nothing is so rapid in its action and so satisfactory as the injection of morphine hypodermically, but we are reminded that morphine lessens the biliary secretion. Antipyrin is not commended, for it is not very efficacious in relieving pain. Turpentine is cited as a remedy, often used in patent medicines, as one of the cholagogues which aid in the expulsion of the gall-stones by inducing a flow of liquid bile; its action, however, is feeble. Sée regards salicylate of sodium and olive oil as the two most powerful remedies; of these the former greatly augmented the watery constituent of the bile, as was shown by Rutherford and other observers, and it acted best when given with water in large quantities. It was also found to lessen the pain, in addition to acting as a cholagogue. Olive oil, by supplying the fatty acids which dissolve the cholesterine, was also a powerful remedy, and this was the physiological explanation of the action of this remedy, which has been given empirically in gall-stones for some time past. Those who are liable to gall-stone should take it regularly, the dislike to it experienced by some patients in commencing its use being soon overcome. Active purgative remedies which induce spasm of the bile-ducts are contra-indicated, as are those drugs which diminish bile secretion, *e.g.*, calomel, iron, copper, and morphine, atropine, strychnine, and alkalies.

The use of olive oil for the relief of hepatic colic was introduced some few years ago, and it appears that this treatment was the outcome of advice given to a physician in the "Wild West" by a village blacksmith. From this Dr. J. Touatre, of New Orleans, was led to try it on himself whilst suffering from gall-stones with acute colic. He proceeded thus: At seven in the evening a blue pill of the weight of fifteen centigrammes was taken, and this was followed twelve-hours later by the taking in one draught of twelve table spoonfuls of olive oil; a quarter of an hour later a similar dose was taken, and then the patient lay down on his right side. At nine o'clock the blue pill acted, producing a copious biliary action, but no gall-stones were passed. At three o'clock in the afternoon another stool was passed without any gall-stones, but in the six stools which came away between seven in the evening and midnight, no less than sixty stones were found, of these seventeen were as large as a pea, and six the size of an olive. The passing of these stones was accomplished with much pain, and was followed by relief from the pre-existing pain. Dr. Touatre was in perfect health for the next three months, when, on a recurrence of the hepatic colic, he once more resorted to the olive oil, and eighteen more gall-stones were passed, since which he had not noticed any discomfort up to the time of his report.

On the other hand, we have reports which seem to prove just as conclusively that the olive oil treatment of gall-stones is absolutely useless. Thus, about ten years ago, Dr. Shingleton Smith recommended a trial of the oil to a fellow medical man who had suffered for years, off and on, from gall-stone colic. He accordingly drank about a tumblerful of oil one morning by sipping it at frequent intervals. In due course the "gall-stones" (?) were passed, but in such very large numbers that Dr. Smith examined them more closely, and found that they were masses of partially saponified oil, easily crumbling on slight pressure, and containing no trace of cholesterine. In this case no benefit resulted from the administration of oil. Again, Mr. A. S. Gubb tells of a medical man, "himself a martyr to the paroxysms, who has given the plan an exhaustive trial, at first with the most promising result, seeing that a copious discharge of calculi (?) followed a dose of the oil. This did not prevent a recurrence of the pain, and this led him to scrutinise the 'stones,' which continued to pass in ridiculous numbers. He was then sorrowfully constrained to admit that the treatment was a snare and a delusion," the calculi being simply concretions of unaltered fatty base.

However, M. Sée lends valuable support to the personal testimony of Dr. Touatre, and we cannot but think that the statements that have been published by some observers that olive oil is absolutely inert in gall-stone colic, and that the so-called gall-stones are nothing more or less than masses of altered oil, require careful investigation before the employment of this method of treatment is given up. One thing seems essential, and this is that the action of the oil must be aided by the exhibition of some remedy such as mercury or salicylate of sodium, which will produce an abundant secretion of bile.

